# Functional Characterization of *ClHSP22.9* Promoter from Wild Watermelon (*Citrullus lanatus*) Reveals Heat- and Drought-Inducible Transcriptional Activity

**DOI:** 10.3390/ijms27219648

**Published:** 2026-10-28

**Authors:** Raheel Munir, Iqbal Hussain, Nazia Jan, Bahar Ali, Iqbal Munir, Waqar Ali, Raham Sher Khan, Aqib Iqbal

**Affiliations:** 1 Department of Horticulture, Zhejiang University, Hangzhou 310058, China; 2 Institute of Biotechnology and Genetic Engineering, The University of Agriculture, Peshawar 25130, Pakistan; 3 Department of Biotechnology, Abdul Wali Khan University, Mardan 23200, Pakistan

**Keywords:** *ClHSP22.9*, wild watermelon, small heat shock protein, stress-responsive promoter, *cis*-regulatory elements, heat stress, drought stress, *Brassica napus*

## Abstract

Small heat shock proteins (sHSPs) are essential components of the plant proteostasis network under environmental stress; however, the *cis*-regulatory mechanisms underlying stress-responsive expression of individual sHSP genes remains insufficiently depicted. Here, we characterized the promoter of *ClHSP22.9* from wild watermelon (*Citrullus lanatus* subsp. *cordophanus*) and evaluated its transcriptional responsiveness under different abiotic stresses. A semi-quantitative RT-PCR analysis revealed that *ClHSP22.9* transcripts were strongly induced by heat, dehydration, and salinity treatments, whereas cold treatment caused comparatively limited transcriptional alterations. A 1482 bp promoter region upstream of the transcription start site was isolated using thermal asymmetric interlaced PCR (TAIL-PCR) and partially overlapping primer-based PCR (POP-PCR). An *in silico* promoter analysis identified multiple predicted stress and hormonal-associated *cis*-regulatory elements, including two heat shock elements (HSEs). Three serial 5′ promoter deletion fragments (−1482, −1012, and −522 bp) were fused to the *GUS* reporter gene and functionally analyzed in transgenic *Brassica napus*. Under heat stress, the −1482 and −1012 bp promoter fragments exhibited strong reporter activation, whereas deletion to −522 bp significantly reduced promoter activity, indicating that the −1012 to −522 bp region contributes substantially to maximal heat-responsive transcription. Furthermore, the −1012 bp proHSPII fragment retained an inducible *GUS* expression under heat, drought, and combined heat and drought stress conditions. These findings identify an important region within the *ClHSP22.9* promoter and establish the −1012 bp promoter fragment as a promising candidate stress-responsive regulatory sequence for further mechanistic characterization and potential application in plant biotechnology.

## 1. Introduction

Plants are continuously exposed to environmental stresses including high temperature, water deficit, salinity, and low temperature, which can disrupt cellular homeostasis and adversely affect growth and productivity. Due to their sessile nature, plants depend on rapid and tightly regulated molecular responses to maintain cellular integrity under unfavorable conditions. An important component of these responses is the induction of heat shock proteins (HSPs), a diverse group of molecular chaperones that accumulate not only during heat stress but also in response to other environmental challenges [1]. Among them, small heat shock proteins (sHSPs/HSP20s) constitute a particularly diverse class of ATP-independent molecular chaperones characterized by a conserved α-crystallin domain. By binding partially unfolded or misfolded proteins, sHSPs limit irreversible protein aggregation and contribute to cellular proteostasis under unfavorable conditions [2,3,4]. Recent analyses further emphasize the functional diversification of plant sHSPs across subcellular compartments and their involvement in responses to heat, drought, salinity, oxidative stress, and other environmental constraints [4].

The stress-responsive accumulation of sHSPs is controlled to a large extent at the transcriptional level. Under heat stress, activated heat shock transcription factors (HSFs) recognize heat shock elements (HSEs) within HSP promoters and stimulate transcription [5]. HSF-dependent regulation is, however, embedded within broader stress-signaling networks, and plant sHSP promoters commonly contain combinations of *cis*-regulatory elements associated with hormonal and environmental responses [6,7,8]. These include ABA-responsive elements (ABREs), MYB-binding sites (MBSs), G-boxes, W-boxes, and other stress-associated motifs. The number, position, orientation, and combinatorial organization of such elements can influence the magnitude, timing, and specificity of promoter output. Recent analyses in *Brassica* similarly identified stress- and hormone-associated *cis*-regulatory elements in promoters of *OSCA* and *PYL* gene families, together with differential transcriptional responses to drought and ABA treatments [9,10]. Thus, the computational identification of *cis*-elements can provide mechanistic hypotheses, but functional promoter analysis is required to determine which regulatory regions are necessary for stress-responsive transcription.

Watermelon HSP20 genes constitute a diverse multigene family and show substantial variation in their transcriptional responses to environmental stimuli. A genome-wide analysis of cultivated watermelon identified numerous HSP20 genes containing the characteristic α-crystallin domain and revealed considerable diversity in their predicted subcellular localization and stress responsiveness [11]. Wild watermelon is particularly relevant for investigating stress-associated molecular responses because of its adaptation to water-limited environments. A previous proteomic analysis of wild-watermelon leaves exposed to a water deficit revealed the extensive accumulation of stress-associated proteins, including several sHSPs [12]. Among these, *CLHSP22.9* was characterized as an endoplasmic-reticulum-type sHSP and showed a pronounced responsiveness to a water deficit [12]. These observations point to the strong environmental regulation of *ClHSP22.9* expression; however, the *cis*-regulatory architecture underlying this responsiveness and the promoter regions required for stress-dependent transcription have not been functionally defined.

The characterization of stress-responsive promoters also has potential value for plant biotechnology. Strong constitutive promoters can maintain a high transgene expression irrespective of environmental conditions, but the sustained expression of stress-associated genes may unnecessarily divert cellular resources or interfere with normal growth and development. Stress-inducible promoters offer an alternative by restricting elevated transgene expression to conditions under which the encoded function is required [13]. Recent advances in inducible-promoter engineering emphasize that promoter performance depends not only on the presence of individual regulatory motifs but also on their organization, transcription-factor interactions, basal activity, induction strength, and environmental specificity [14]. The functional characterization of native stress-responsive promoters through deletion analysis and reporter assays can therefore provide both mechanistic information about transcriptional regulation and candidate regulatory sequences for environmentally responsive expression systems.

In the present study, we investigated the stress-responsive transcription of *ClHSP22.9* in wild watermelon and functionally characterized its upstream regulatory region. We first examined native *ClHSP22.9* transcript accumulation under heat, dehydration, salinity, and cold treatments. A 1482 bp sequence upstream of the transcription start site was subsequently isolated and analyzed for putative *cis*-regulatory elements. To functionally restrict promoter regions contributing to heat responsiveness, the full-length promoter and two serial 5′ deletion fragments (−1482, −1012, and −522 bp relative to the transcription start site) were fused to a *GUS* reporter and evaluated in transgenic *Brassica napus*. The −1012 bp fragment was further assessed under heat, drought, and combined heat/drought treatments. This integrated approach was designed to define regulatory regions contributing to *ClHSP22.9* stress-responsive transcription and to evaluate whether a truncated promoter fragment retains multi-stress-responsive activity in a heterologous plant system.

## 2. Results

### 2.1. ClHSP22.9 Exhibits Distinct Transcriptional Responses to Abiotic Stresses in Wild Watermelon

**Figure 1:**
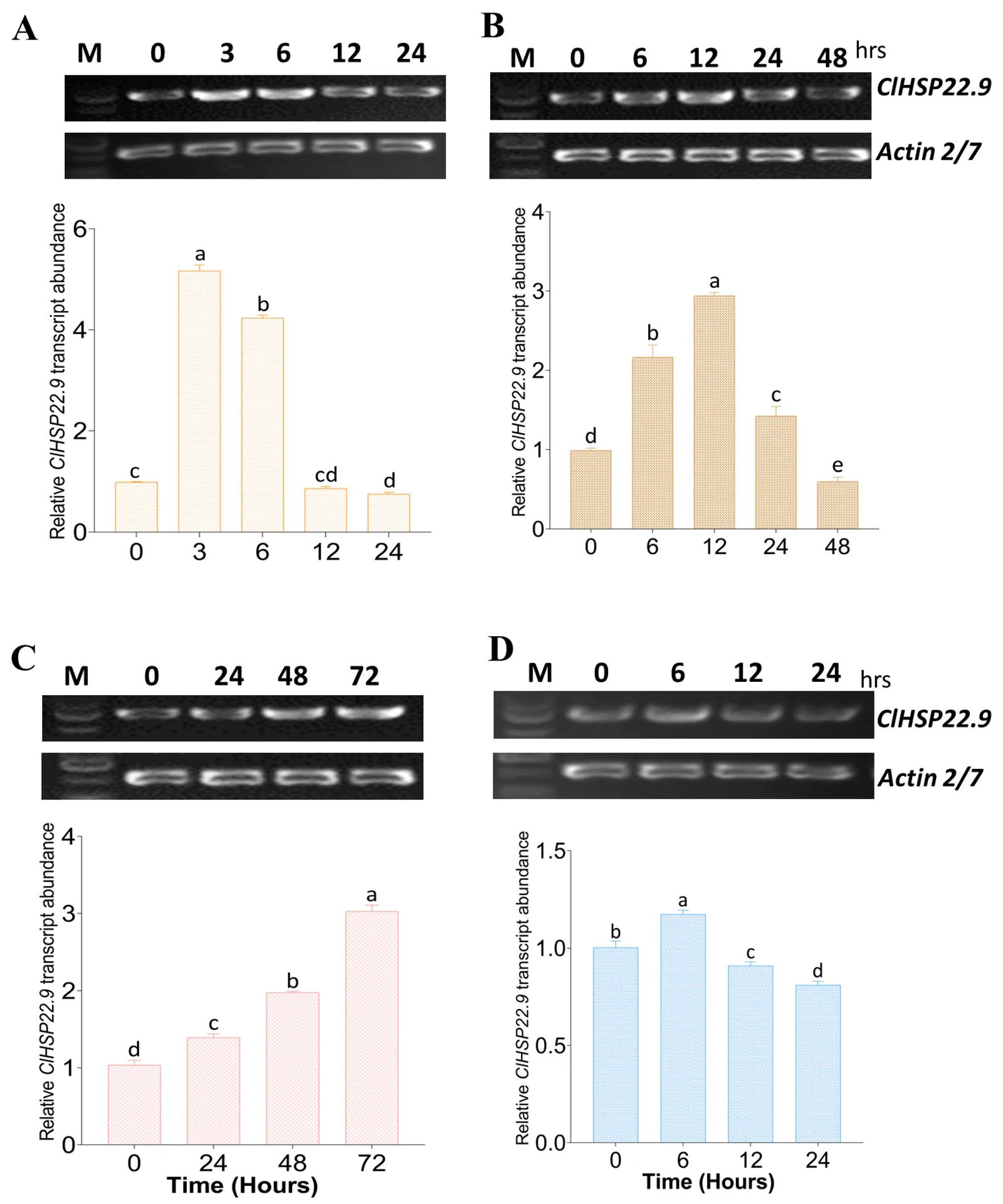
Abiotic stress-responsive expression of *ClHSP22.9* in wild watermelon. Semi-quantitative RT-PCR analysis of *ClHSP22.9* transcript accumulation in wild-watermelon (*Citrullus lanatus*) leaves exposed to different abiotic stresses. Plants were exposed to (**A**) heat stress (42 °C) for 0, 3, 6, 12, and 24 h; (**B**) dehydration stress for 0, 6, 12, 24, and 48 h; (**C**) salt stress (200 mM NaCl) for 0, 24, 48, and 72 h; and (**D**) cold stress (4 °C) for 0, 6, 12, and 24 h. Representative RT-PCR products are shown above the corresponding densitometric analyses. *Actin 2/7* was used as the internal reference gene. Relative transcript abundance of *ClHSP22.9* was calculated by normalizing band intensity to the corresponding *Actin 2/7* signals and expressing values relative to the respective 0 h control. Data are presented as mean ± SD (n = 3 biological replicates). Different lowercase letters indicate significant differences among time points within each stress treatment according to one-way ANOVA, followed by Tukey’s HSD multiple-comparison test (*p* < 0.05). M = molecular size marker.

To determine *ClHSP22.9* response against different abiotic stresses, its transcript abundance was examined in wild-watermelon leaves exposed to heat, dehydration, salinity, and low temperature using semi-quantitative RT-PCR (Figure 1). The optimization of the amplification cycle number showed that 30 cycles provided readily detectable *ClHSP22.9* amplification while remaining suitable for semi-quantitative analysis (Figure S2). *Actin 2/7* was used as the internal reference, and band intensities were quantified by densitometric analysis.

Heat treatment at 42 °C caused a rapid and pronounced induction of *ClHSP22.9*. Transcript abundance increased to approximately 5.2-fold relative to the untreated control at 3 h and remained strongly elevated at 6 h (~4.2-fold), before declining substantially at 12 and 24 h (Figure 1A). Thus, the heat response was characterized by a rapid but transient accumulation of *ClHSP22.9* transcripts, whereas a comparatively gradual response was observed following the dehydration of excised leaves, with transcript abundance increasing progressively to approximately 2.2-fold at 6 h and a maximum of ~2.9-fold at 12 h. The expression subsequently declined at 24 h and fell below the initial level after 48 h of dehydration (Figure 1B). Similarly, NaCl treatment also induced *ClHSP22.9*, although with slower kinetics. Exposure to 200 mM NaCl resulted in a gradual increase in relative transcript abundance over the treatment period, reaching approximately threefold at 72 h (Figure 1C). In contrast, cold treatment at 4 °C caused only modest changes: a small transient increase was observed at 6 h, followed by a gradual decrease at 12 and 24 h (Figure 1D). Overall, these results indicate that *ClHSP22.9* is responsive to multiple abiotic stresses, with the strongest and more rapid transcriptional induction occurring under heat stress. The differences among time points within each treatment were evaluated by one-way ANOVA, followed by Tukey’s HSD test (*p* < 0.05). Cold and salinity treatments were included in this initial expression analysis to assess the broader abiotic stress responsiveness of endogenous *ClHSP22.9*, whereas subsequent promoter-functionalization experiments focused on heat and drought responses.

### 2.2. Isolation and Cis-Regulatory Characterization of the ClHSP22.9 Promoter

**Figure 2:**
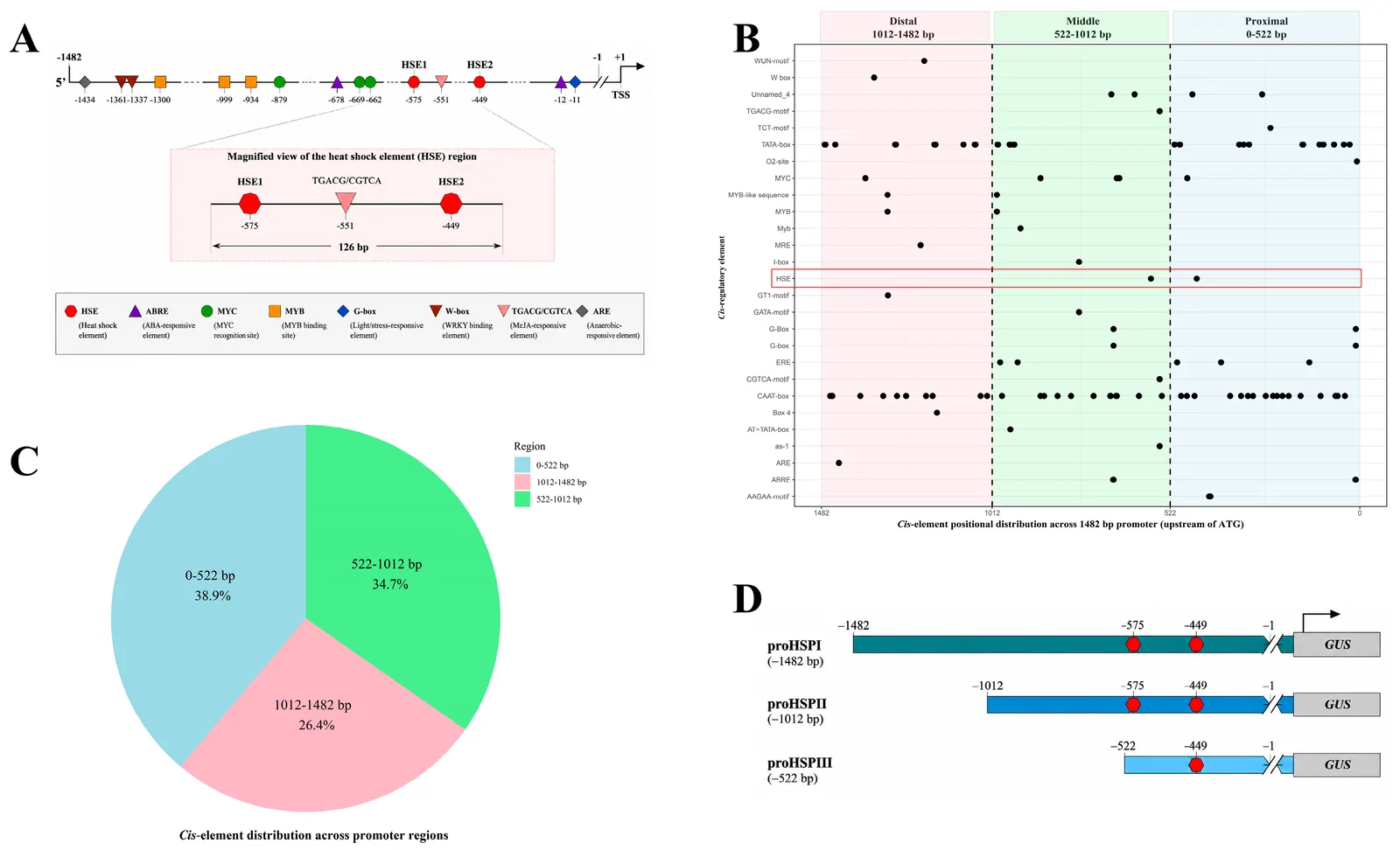
*Cis*-regulatory architecture and construction of serial 5′ deletion fragments of the *ClHSP22.9* promoter. (**A**) Distribution of predicted *cis*-regulatory elements within the 1482 bp promoter region, with an enlarged view of the two predicted heat shock elements (HSE1 and HSE2). Symbols represent the different *cis*-element classes, as indicated in the legend. (**B**) Positional distribution of predicted *cis*-regulatory elements across the distal (−1482 to −1012 bp), middle (−1012 to −522 bp), and proximal (−522 to −1 bp) promoter regions. (**C**) Percentage distribution of *cis*-regulatory elements among the three regions. (**D**) Schematic representation of the promoter::GUS constructs used for functional analysis in transgenic *Brassica napus*: proHSPI (−1482 bp), proHSPII (−1012 bp), and proHSPIII (−522 bp). The first two constructs retain both predicted HSEs (−575 and −449 bp), whereas proHSPIII retains only the HSE at −449 bp. Promoter coordinates are defined relative to the reference position used in this study.

The assembled sequence yielded a 1482 bp upstream region of *ClHSP22.9*, which was used for *cis*-regulatory analysis and functional characterization (Figure S1; Tables S2–S4). An *in silico* analysis identified 36 classes of putative *cis*-regulatory elements within this region (Table S5), including motifs associated with environmental stress responses, hormonal signaling, developmental regulation, and light responsiveness. The predicted elements included heat shock elements (HSEs), MYB-binding sites (MBSs), ABA-responsive elements (ABREs), anaerobic-responsive elements (AREs), low-temperature-responsive elements (LTRs), W-boxes, G-boxes, and TGACG/CGTCA motifs associated with methyl jasmonate responsiveness (Figure 2A). Among these motifs, two predicted HSEs were identified at approximately −575 bp (HSE1) and −449 bp (HSE2) relative to the reference transcription start site (TSS). An enlarged view of the HSE-containing region revealed that both elements are located within a 126 bp promoter interval, suggesting a potentially important regulatory region associated with heat-responsive transcription (Figure 2A). To further characterize the spatial organization of *cis*-regulatory elements, the promoter region was divided into three sections relative to the TSS: the distal region (−1482 to −1012 bp), middle region (−1012 to −522 bp), and proximal region (−522 to −1 bp). The distribution analysis revealed that the predicted *cis*-elements were not evenly distributed across the promoter, with elements detected throughout all three regions (Figure 2B). The quantitative representation of *cis*-element abundance showed that the proximal region contained the highest proportion of predicted regulatory elements (38.8%), followed by the middle (34.7%) and distal regions (26.4%) (Figure 2C), indicating that the regulatory information is distributed across the promoter rather than confined to a single upstream region. Moreover, based on the promoter architecture and the positions of predicted HSEs, three serial 5′ deletion fragments were generated for functional promoter analysis: the full-length 1482 bp promoter fragment (proHSPI), the intermediate 1012 bp fragment (proHSPII), and the shortest 522 bp fragment (proHSPIII) (Figure 2D). The proHSPI and proHSPII fragments retained both predicted HSEs located at approximately −575 and −449 bp, whereas the proHSPIII fragment retained only the proximal HSE at −449 bp relative to the TSS. Each promoter fragment was subsequently fused upstream of the *GUS* reporter gene for functional evaluation in transgenic *Brassica napus*.

### 2.3. Generation and Molecular Confirmation of ClHSP22.9 Promoter::GUS Transgenic Plants

**Figure 3:**
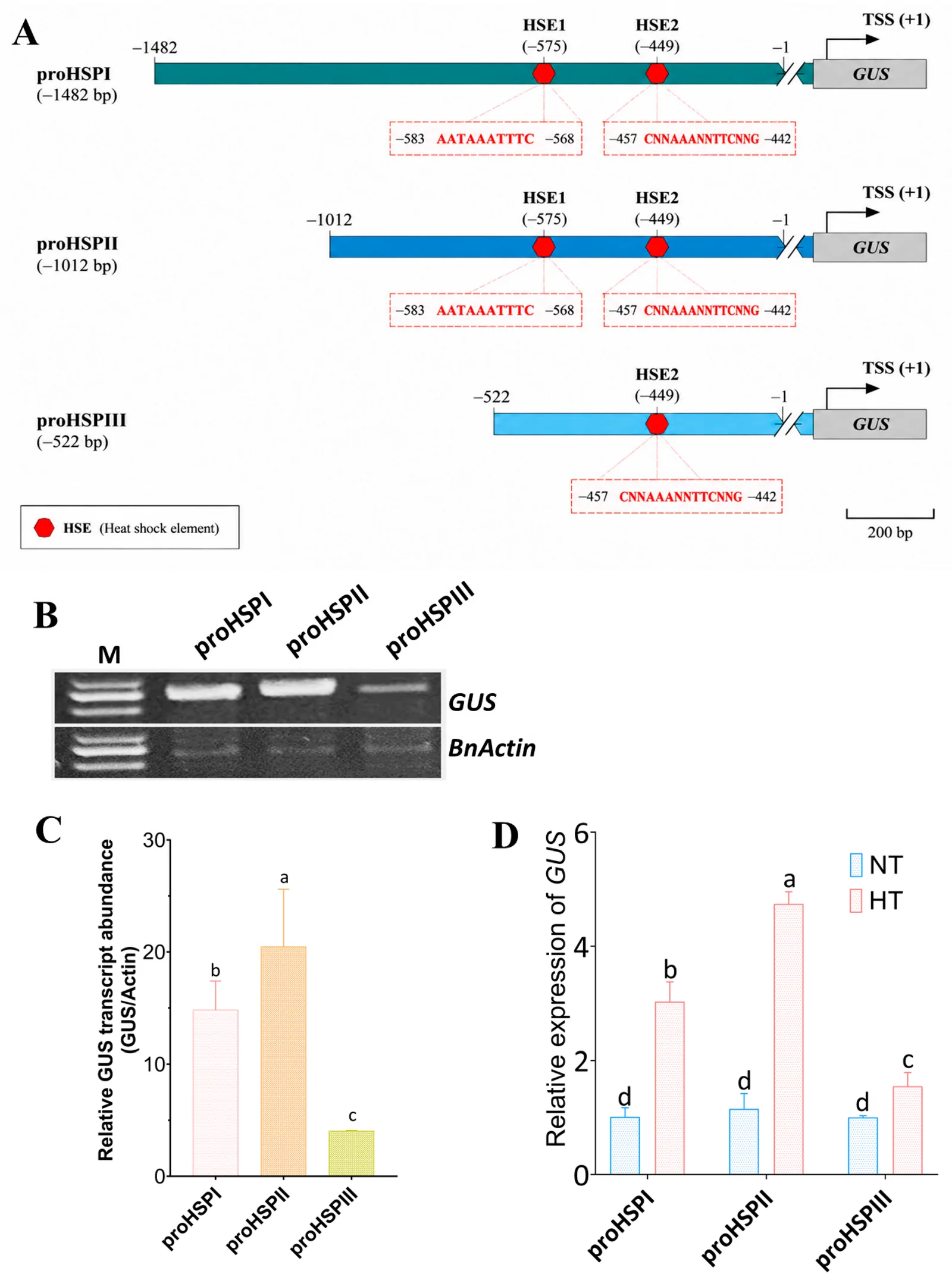
Functional analysis of *ClHSP22.9* promoter regions associated with heat-responsive activity. (**A**) Schematic representation of the three serial deletion fragments of the *ClHSP22.9* promoter fused upstream of the *GUS* reporter gene. The full-length promoter fragment, proHSPI (−1482 bp), and the intermediate fragment, proHSPII (−1012 bp), contain two predicted heat shock elements (HSEs) located approximately at −575 bp and −449 bp relative to the TSS (+1), whereas the shortest fragment, proHSPIII (−522 bp), retains only the proximal HSE at approximately −449 bp. The sequences of the predicted HSE regions are indicated below the corresponding promoter constructs. (**B**) Representative semi-quantitative RT-PCR analysis of *GUS* and *Actin* transcripts in transgenic *B. napus* plants harboring the three promoter::*GUS* constructs (proHSPI::*GUS*, proHSPII::*GUS*, and proHSPIII::*GUS*) under heat stress conditions. M, molecular size marker. (**C**) Densitometric quantification of *GUS* transcript abundance normalized against the corresponding *Actin* signal. (**D**) Quantitative real-time PCR (RT-qPCR) analysis of GUS transcript expression in transgenic plants carrying proHSPI, proHSPII, and proHSPIII under normal, unstressed conditions (NT) and heat stress (HT). Data are presented as mean ± SD (n = 3 biological replicates). Different lowercase letters indicate significant differences among groups, as determined by one-way ANOVA, followed by Tukey’s HSD multiple-comparison test (*p* < 0.05) for (**C**), and by two-way ANOVA, followed by Tukey’s HSD multiple-comparison test for (**D**) (*p* < 0.05).

PCR screening confirmed the presence of the GUS transgene in 18 of 25 T_2_ plants carrying the proHSPII::*GUS* construct (72%; Figure S5). Quantitative RT-PCR further demonstrated differences in *GUS* transcript abundance among the three promoter constructs under heat-stress conditions and between unstressed and heat-stressed conditions for the selected constructs (Figure 3). These findings support the transcriptional responsiveness of the promoter fragments, although direct *GUS* enzymatic activity was not assessed.

### 2.4. Serial 5′ Deletion Analysis Identifies an Upstream Region Contributing to Full Heat-Responsive Promoter Activity

To determine how progressive 5′ deletion affected *ClHSP22.9* promoter activity, transgenic *B. napus* plants carrying proHSPI::*GUS*, proHSPII::*GUS*, or proHSPIII::*GUS* were subjected to heat treatment at 42 °C. An initial heat treatment time-course was performed at 0, 3, 6, 12, and 24 h, and the strongest *GUS* transcript accumulation was observed at 6 h. Therefore, the 6 h time point was selected for a comparative analysis of the three promoter fragments (Figure 3).

Semi-quantitative RT-PCR showed strong *GUS* transcript accumulation in plants carrying either the 1482 bp proHSPI or the 1012 bp proHSPII fragment. In contrast, the truncation of the promoter to 522 bp resulted in markedly lower *GUS* transcript abundance (Figure 3B,C). Thus, the deletion of the distal promoter region substantially reduced reporter transcript accumulation, whereas the proHSPII (−1012 bp) fragment retained strong promoter activity. The differences among the three promoter constructs were evaluated by one-way ANOVA, followed by Tukey’s HSD test (*p* < 0.05). To further quantify the reporter-gene expression and assess basal promoter activity, RT-qPCR was performed for all three promoter constructs under unstressed control and heat-stress conditions (Figure 3D). Under unstressed conditions, *GUS* transcript abundance was comparable among proHSPI, proHSPII, and proHSPIII. Following heat treatment, *GUS* transcript abundance increased, with proHSPII exhibiting the highest expression among the three constructs. These quantitative results support the heat-responsive transcriptional activity of the promoter fragments and further indicate that the −1012 bp proHSPII fragment provides the strongest reporter-transcript response among the constructs tested. The RT-qPCR data were analyzed using two-way ANOVA followed by post hoc multiple-comparison testing (*p* < 0.05). The proHSPIII deletion removes the promoter region between −1012 and −522 bp while retaining the proximal predicted HSE at approximately −449 bp. In contrast, proHSPII retains both predicted HSEs located at approximately −575 and −449 bp (Figure 3A). The pronounced reduction in reporter expression following truncation to −522 bp indicates that sequence(s) within the −1012 to −522 bp interval contribute substantially to full heat-responsive promoter activity. Although this interval contains the distal predicted HSE, the deletion experiment does not establish that this element alone accounts for the observed reduction in promoter activity, as other regulatory sequences within the deleted region may also contribute. The site-directed mutagenesis of the individual HSEs, alone or in combination, would help clarify their specific contributions to heat-responsive promoter activity.

### 2.5. The proHSPII Fragment Confers Stress-Responsive Reporter Expression in Transgenic B. napus

**Figure 4:**
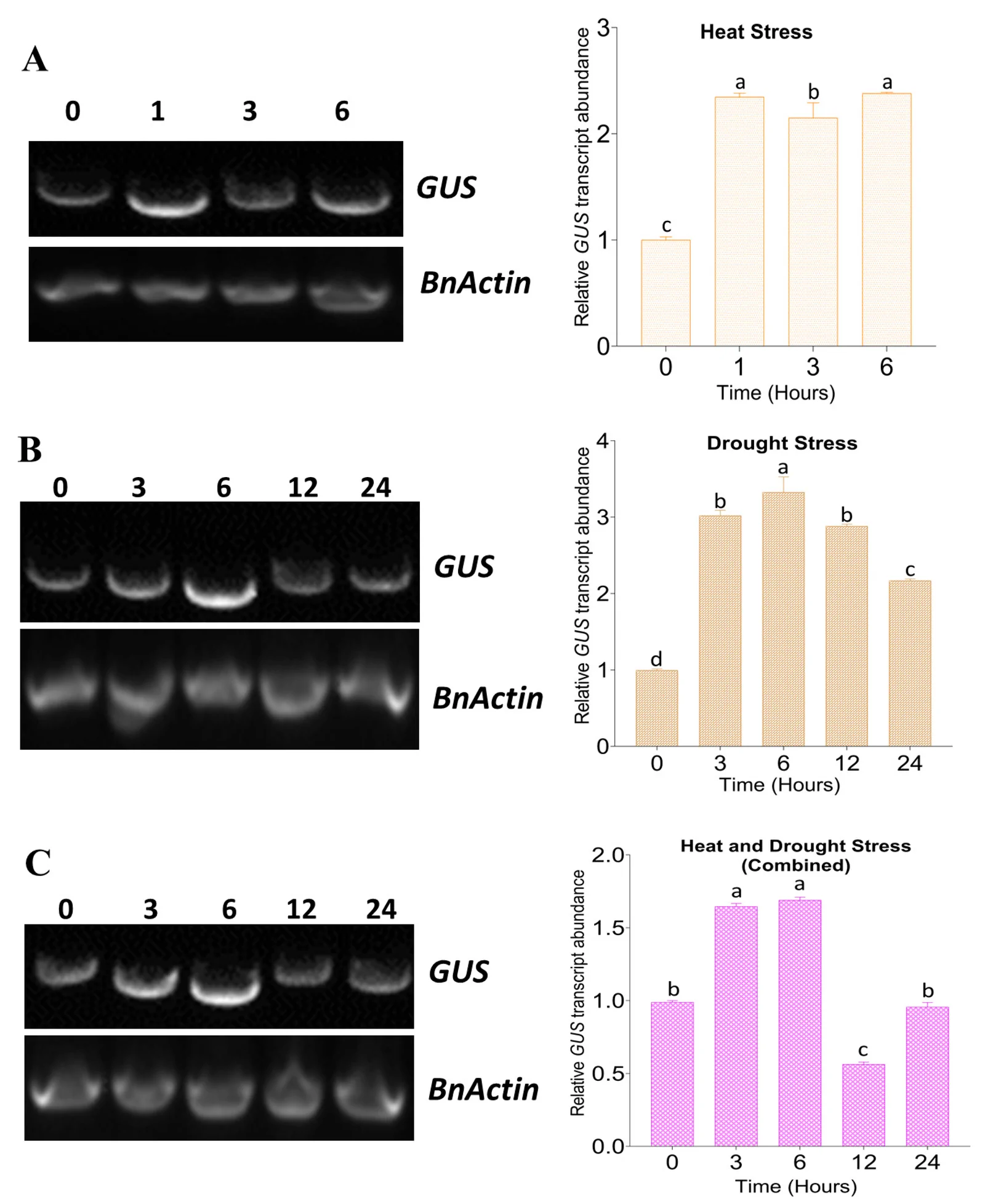
Stress-responsive activity of the −1012 bp *ClHSP22.9* promoter in transgenic *Brassica napus*. Semi-quantitative RT-PCR analysis of *GUS* reporter expression in transgenic *B. napus* plants harboring the proHSPII:*GUS* construct following exposure to (**A**) heat stress at 42 °C for 0, 1, 3, and 6 h, (**B**) drought stress for 0, 3, 6, 12, and 24 h, and (**C**) combined heat and drought stress for 0, 3, 6, 12, and 24 h. Representative RT-PCR products are shown together with corresponding densitometric analyses. *Actin* was used as the internal reference gene. Relative *GUS* transcript abundance was calculated by normalizing band intensity to the corresponding *Actin* signal and expressing values relative to the respective 0 h controls. Data are presented as mean ± SD (n = 3 biological replicates). Different lowercase letters indicate significant differences among time points within each treatment according to one-way ANOVA, followed by Tukey’s HSD multiple-comparison test (*p* < 0.05).

The proHSPII fragment (−1012 bp) retained strong heat-responsive activity despite the deletion of the distal promoter region. Therefore, the proHSPII fragment was selected for further functional evaluation under heat, drought, and combined heat–drought treatments (Figure 4). Exposure to 42 °C rapidly induced reporter expression, with *GUS* transcript abundance increasing within 1 h and remaining elevated at 3 and 6 h (Figure 4A). These results demonstrate the rapid activation of the proHSPII promoter fragment in response to heat stress. Furthermore, the drought treatment also induced proHSPII activity, although with different temporal kinetics. *GUS* transcript abundance increased by 3 h, reached its highest level at approximately 6 h, and subsequently declined at 12 and 24 h while remaining above the untreated level (Figure 4B), suggesting that the −1012bp promoter fragment also responds to water-deficit conditions. Interestingly, the combined heat and drought treatment similarly induced *GUS* transcript accumulation, with reporter expression increasing at 3 h and reaching its highest or near-highest level at approximately 6 h. Transcript abundance subsequently declined at 12 and 24 h (Figure 4C), indicating a predominantly transient transcriptional response of the promoter under simultaneous heat and drought stress. Collectively, these results demonstrate that proHSPII fragment (−1012 bp) retains inducible reporter activity under heat, drought, and simultaneous heat and drought stress.

## 3. Discussion

Small heat shock proteins form a highly responsive component of the plant proteostasis network; yet, individual sHSP genes differ considerably in the environmental cues that activate their transcription and in the regulatory architecture that underlies those responses [1,2,3,4]. The present study links the stress-responsive expression of *ClHSP22.9* with the functional organization of its promoter. *ClHSP22.9* exhibited distinct transcriptional responses to heat, dehydration, salinity, and cold, while serial promoter deletion analysis identified a substantial component of heat-responsive activity within the −1012 to −522 bp region. Importantly, the −1012 bp (proHSPII) fragment retained inducible activity after the transfer into *Brassica napus*, indicating that regulatory information encoded within this region can be interpreted by the transcriptional machinery of a heterologous dicot species. Together, these findings establish a functional association between promoter-region composition and stress-responsive transcription, although the contribution of individual *cis*-regulatory elements need to be resolved.

The rapid induction of *ClHSP22.9* by heat is consistent with the dynamic transcriptional behavior reported for plant HSP20 genes, many of which respond rapidly to elevated temperature. Genome-wide studies in several plant species have similarly shown substantial variation among HSP20 family members in the magnitude and timing of responses to heat, drought, salinity, osmotic stress, and cold [15,16,17,18]. Such temporal variation is biologically relevant because heat-shock responses must be rapidly activated and subsequently attenuated as plants acclimate to changing temperature conditions. Recent work emphasizes that HSF activity is dynamically regulated rather than functioning as a simple on/off response, with timely attenuation contributing to the restoration of cellular homeostasis [19]. The comparatively gradual accumulation of *ClHSP22.9* transcripts following the dehydration of excised leaves suggests that the gene can also respond to water-deficit-related signals through kinetics distinct from those observed during heat treatment. An important limitation of the native expression analysis is that the stress treatments were performed using excised leaves rather than intact plants. Leaf detachment may alter water relations and stress-signaling processes; therefore, the observed transcriptional responses characterize excised leaf tissues under the applied treatment conditions. These responses are consistent with previous observations in wild watermelon, in which a water deficit caused strong sHSP accumulation and increased *CLsHSP22.9* transcript abundance [12]. The present results extend these observations by comparing the temporal expression patterns of *ClHSP22.9* under several abiotic stress treatments. A further investigation of the underlying signaling mechanisms would provide additional insights into the regulation of *ClHSP22.9* expression under these conditions.

The *cis*-regulatory landscape of the 1482 bp promoter provides a plausible basis for this multi-stress responsiveness. In addition to two predicted HSEs, the promoter contains ABREs, MBSs, W-boxes, G-boxes, LTRs, TGACG/CGTCA motifs, and other regulatory elements associated with environmental and hormonal signaling. HSEs are recognized by heat shock transcription factors and constitute canonical regulatory elements of heat-inducible genes [5]. However, the presence of an HSE motif alone does not establish its functionality, and promoter output depends on the sequence, number, spacing, orientation, chromatin context, and availability of the corresponding transcription factors. This distinction is particularly relevant to the present study because the promoter also contains motifs associated with ABA, MYB, WRKY, light, and jasmonate-related regulation. Earlier studies in *Brassica* have likewise associated predicted promoter *cis*-regulatory elements with stress and hormone-responsive expression [9,10,20]. The limited transcriptional response of *ClHSP22.9* to cold despite the presence of a predicted LTR further illustrates why computational motif prediction should not be equated with functional regulatory activity. *Cis*-element prediction identifies candidate regulatory sites, whereas their contribution to transcription depends on the surrounding sequence context and the cellular signaling state. A similar divergence between the predicted promoter architecture and experimentally observed expression has been reported for HSP20 genes in other plant species [15,16,17,18]. Thus, the *cis*-regulatory analysis in the present study provides a framework for interpreting promoter behavior but does not by itself establish the activity of individual motifs.

The serial 5′ deletion analysis provides the strongest functional evidence for the regulatory organization of the promoter. The −1482 bp proHSPI and −1012 bp proHSPII fragments retained strong reporter expression following heat treatment, whereas deletion to −522 bp resulted in a pronounced reduction in *GUS* transcript accumulation. Notably, proHSPIII retains the proximal HSE at −449 bp; yet, its reporter activity was substantially lower than that of the longer promoter fragments. These observations indicate that the −449 bp HSE alone is insufficient to reproduce the activity of the longer promoter constructs. The deleted −1012 to −522 bp interval contains the second predicted HSE at −575 bp, together with additional predicted regulatory elements. Therefore, this interval represents an important candidate regulatory region contributing to maximal heat-responsive promoter activity. Importantly, the present deletion experiment cannot attribute the reduced activity specifically to the −575 bp HSE, because approximately 490 bp of the promoter sequence is removed simultaneously. The observed reduction could result from the loss of the HSE, one or more neighboring regulatory elements, altered interactions among transcription-factor binding sites, or changes in promoter spacing and architecture. Previous promoter analyses support such combinatorial regulation. For example, studies of plant sHSP promoters have shown that HSEs can function in conjunction with neighboring regulatory sequences to achieve strong heat-induced transcription [21,22,23]. The combined analysis of *GUS* transcript abundance with histochemical and fluorometric *GUS* assays to characterize the wheat sHSP26 promoter illustrates how transcript-level measurements can be complemented by direct reporter-enzyme activity measurements [23]. Similarly, another study demonstrated that the targeted mutagenesis of an imperfect HSE in the sunflower *Ha hsp17.6 G1* promoter impaired reporter expression during seed development, while deletion analysis identified additional upstream sequences contributing to its regulation [22]. These studies illustrate the complementary value of quantitative reporter assays and targeted *cis*-element mutagenesis in distinguishing promoter activity from the specific regulatory mechanisms underlying it. Accordingly, the site-directed mutation of the −575 bp and/or −449 bp HSE within an otherwise intact promoter would provide a more direct test of their individual contributions than further large-scale deletion analysis.

The ability of proHSPII to function in transgenic *B. napus* provides additional evidence that the −1012 bp fragment contains regulatory information that can be recognized outside its native species. Similar cross-species promoter activity has been reported for several plant stress-responsive promoters, including the wheat sHSP26 promoter in transgenic *Arabidopsis* and the *Arabidopsis* GRPL1 promoter in heterologous systems [24,25,26]. Nevertheless, reporter activity in a heterologous system does not establish that the promoter behaves identically in its native species, because transgene insertion position, copy number, chromatin environment, and species-specific transcription-factor abundance can influence reporter expression. Therefore, the present assay demonstrates the functional portability of the promoter region rather than the complete conservation of its native regulatory context. A particularly relevant finding is that the proHSPII fragment remained responsive not only to heat but also to drought stress conditions. Plants frequently encounter multiple environmental constraints simultaneously, and combined heat and drought can produce transcriptional states that differ from those induced by either stress alone. Multi-omics analyses have shown that combined heat and drought can involve stress-specific transcriptional programs rather than a simple additive combination of individual responses [27,28,29,30]. Recently, a transcriptomic analysis in crop species indicate that combined heat and drought can generate molecular responses that differ substantially from those observed under individual stress conditions [19,31]. The transient activation of proHSPII under combined heat and drought therefore demonstrates that the promoter remains inducible under a more complex environmental condition. The subsequent decline in reporter transcript abundance may reflect transcriptional attenuation, acclimation, altered transcription-factor activity, or increasing physiological limitation. 

From an applied molecular perspective, the retention of stress-responsive activity within a relatively compact 1012 bp promoter fragment is of potential interest, because environmentally inducible promoters can provide greater temporal control of transgene expression than constitutive promoters and potentially reduce the physiological burden associated with continuous high-level expression [13]. Functional and synthetic promoter studies have shown that relatively compact regulatory sequences can be engineered or selected to provide water-deficit, salt, or heat-responsive expression, and that promoter performance is strongly influenced by the identity, copy number, spacing, and arrangement of *cis*-regulatory elements [32,33,34]. Nevertheless, proHSPII should presently be regarded as a candidate stress-inducible regulatory sequence rather than a fully characterized promoter. Its basal activity under non-stress conditions, tissue and developmental specificity, dynamic induction range, response to stress intensity, and stability across independent transgenic events remain to be determined. Although the additional RT-qPCR analysis strengthens the quantitative assessment of *GUS* transcript abundance, direct histochemical and fluorometric *GUS* assays would provide complementary evidence of reporter activity. Furthermore, the targeted mutagenesis of individual and combined HSEs, followed where appropriate by transcription-factor binding assays, will be necessary to establish the specific contributions of these elements and other predicted *cis*-regulatory motifs to stress-responsive promoter activity. An analysis of additional independent transgenic lines would also strengthen the assessment of reproducibility. Given the specific objectives of the present study, the effects of proHSPII on the physiological responses of plants under stress conditions were not investigated, representing a limitation of the current work. Furthermore, although endogenous *ClHSP22.9* expression was examined under cold and salinity treatments, promoter::*GUS* activity was not evaluated under these conditions; therefore, the responsiveness of the identified promoter fragments to cold and salinity remains to be established. Despite these limitations, the present study identifies the −1012 to −522 bp interval as an important contributor to heat-responsive *ClHSP22.9* promoter activity and demonstrates that the proHSPII (−1012 bp) fragment retains inducible transcriptional activity under heat, drought, and their simultaneous stress. These findings provide a functional basis for the further dissection of the *ClHSP22.9* promoter and establish proHSPII as a promising candidate regulatory sequence for the subsequent characterization of stress-inducible transcription.

## 4. Materials and Methods

### 4.1. Plant Material and Stress Treatments

Wild watermelon (*Citrullus lanatus* subsp. *Cordophanus*) plants used in this study were collected from field locations in Karak and Kohat, Khyber Pakhtunkhwa, Pakistan, and transferred to the greenhouse at the Institute of Biotechnology and Genetic Engineering, The University of Agriculture, Peshawar. Plants of approximately similar size and at the vegetative stage were selected for the experiments and maintained under normal greenhouse conditions for approximately 5–7 days to allow acclimation before stress treatment. Plant material was used for genomic DNA and total RNA isolation. The genomic and promoter sequences of *ClHSP22.9* used for primer design and promoter characterization were obtained from the corresponding watermelon genomic sequence. The corresponding gene locus is annotated as *Clc02G06890* in CuGenDBv2. The reference genome of *Citrullus lanatus* subsp. *cordophanus* is available under GenBank accession JADPLL000000000 (assembly version JADPLL010000000). The upstream genomic region used for promoter amplification was identified from the annotated watermelon genomic sequence and used for subsequent promoter characterization. Promoter coordinates were defined relative to this reference position, and the upstream genomic region was used for promoter amplification and subsequent characterization.

To assess the native transcriptional response of *ClHSP22.9* to abiotic stress, fresh leaves were collected from greenhouse-grown wild watermelon plants following the acclimation and subjected *in vitro* to heat, dehydration, salinity, or low-temperature treatments. In this study, *in vitro* refers to the treatment of excised leaves outside the intact plant. For heat stress, leaves were incubated at 42 °C and sampled at 0, 3, 6, 12, and 24 h. Dehydration was imposed by incubating excised leaves at room temperature, with samples collected at 0, 6, 12, 24, and 48 h. For salinity treatment, the entire blade of each excised leaf was immersed in 200 mM NaCl solution, and sampled at 0, 24, 48, and 72 h. For low-temperature treatment, leaves were incubated at 4 °C and sampled at 0, 6, 12, and 24 h. Samples were immediately frozen in liquid nitrogen and stored at −80 °C until RNA extraction. Three biological replicates were analyzed for each treatment and sampling time.

### 4.2. Total RNA Extraction and Semi-Quantitative RT-PCR Analysis

Total RNA was isolated from treated and untreated leaf samples using a commercial total RNA extraction kit (Thermo Fisher Scientific, Waltham, MA, USA) according to the manufacturer’s instructions. First-strand cDNA was synthesized from 2 μg of total RNA using an oligo(dT)20 primer and Moloney Murine Leukemia Virus (M-MLV) reverse transcriptase (Thermo Fisher Scientific, USA). *ClHSP22.9* transcripts were amplified using gene-specific forward and reverse primers. *Actin 2/7* (GenBank accession GU565958) was used as the internal reference. Primer sequences and corresponding applications are provided in Table S1.

PCR amplification was initiated at 94 °C for 2 min, followed by 30 cycles of 94 °C for 30 s, 55 °C for 30 s, and 72 °C for 1 min using 2.5 U of Taq DNA polymerase. To determine an amplification cycle number appropriate for semi-quantitative analysis, PCR products were evaluated after 25, 30, 35, and 40 cycles. Thirty cycles provided readily detectable amplification while maintaining suitability for comparative densitometric analysis and were therefore used for subsequent experiments (Figure S2).

PCR products were separated by agarose gel electrophoresis, and band intensities were quantified by densitometric analysis. *ClHSP22.9* signal intensity was normalized to the corresponding *Actin 2/7* signal. For individual stress-response time courses, normalized transcript abundance was expressed relative to the corresponding untreated 0 h sample.

### 4.3. Isolation of the ClHSP22.9 Promoter by TAIL-PCR and POP-PCR

Genomic DNA extracted from wild watermelon was used to isolate the upstream regulatory region of *ClHSP22.9*. An initial upstream fragment was obtained by thermal asymmetric interlaced PCR (TAIL-PCR) using nested gene-specific primers in combination with arbitrary primers, following the principle described by Liu and Whittier [35]. Three arbitrary primers (AD1-AD3) and three nested *ClHSP22.9*-specific primers (HSP22.1, HSP22.2, and HSP22.3) were used. Primer sequences and the complete TAIL-PCR cycling program are provided in Tables S2 and S3, respectively. Tertiary amplification products were resolved by agarose gel electrophoresis, purified, and sequenced. This initial genome-walking step yielded approximately 500–575 bp of sequence upstream of *ClHSP22.9*. 

The upstream sequence was subsequently extended by partially overlapping primer-based PCR (POP-PCR) according to the genome-walking strategy described by Li et al. [36]. Three POP primer sets were used, each consisting of primary, secondary, and tertiary primers design according to the established overlapping-primer strategy. Two nested sequence-specific primers, proHSP.G1 and proHSP.G2, were designed from the promoter sequence obtained in the initial genome-walking step. Primer sequences are listed in Table S2, and the corresponding thermal cycling conditions are provided in Table S4.

Primary POP-PCR reactions were performed in a final volume of 50 μL containing 1× PCR buffer, 0.4 mM dNTPs, 0.2 μM of each primer, approximately 100 ng genomic DNA, and 2.5 U Taq DNA polymerase. For secondary and tertiary amplification, 1 μL of the preceding PCR product was used as template under the reaction conditions specified in Table S4. Tertiary PCR products were gel-purified and subjected to Sanger sequencing by Macrogen Inc. (Seoul, Republic of Korea) for sequence confirmation and assembly of the *ClHSP22.9* upstream regulatory region. Overlapping sequences obtained from TAIL-PCR and successive POP-PCR reactions were assembled to reconstruct the upstream regulatory region. The final promoter sequence used for subsequent analyses comprised 1482 bp upstream of the transcription start site (TSS; +1).

### 4.4. In Silico Analysis of Cis-Regulatory Elements

The isolated 1482 bp *ClHSP22.9* promoter sequence was analyzed for putative *cis*-regulatory elements using the PLACE [37] and PlantCARE [33] databases. Predicted elements were classified according to their reported associations with core transcriptional regulation, environmental stress responses, hormonal signaling, developmental processes, or light responsiveness.

The complete inventory of predicted *cis*-elements, including motif sequence, position, strand orientation, copy number, and reported regulatory association, is provided in Table S5. Selected stress and hormone-associated motifs including heat shock elements (HSEs), MYB-binding sites (MBSs), ABA-responsive elements (ABREs), anaerobic-responsive elements (AREs), low-temperature-responsive elements (LTRs), W-boxes, G-boxes, and TGACG/CGTCA motifs were used to generate the promoter map illustrated in Figure 2A.

### 4.5. Construction of Serial 5′ Promoter Deletions and GUS Reporter Vectors

Three promoter fragments extending from −1482, −1012, and −522 bp to the TSS (+1) were amplified and designated proHSPI, proHSPII, and proHSPIII, respectively. Forward primers introduced a HindIII restriction site, whereas the common reverse primer introduced a BamHI site. Primer sequences are provided in Table S6. The amplified promoter fragments were initially cloned into pUC57 for amplification and sequence verification. Sequence-verified promoter inserts were subsequently transferred upstream of the *β*-glucuronidase (*GUS/uidA*) reporter gene, a widely used transcriptional reporter in plants [38], in the binary vector pIG121.Hm (GenBank accession AB489142). The pIG121.Hm system contains an intron-containing *GUS* reporter designed for plant-cell-specific expression [39]. The constitutive CaMV 35S promoter was removed from pIG121.Hm by HindIII/BamHI digestion and replaced with the corresponding *ClHSP22.9* promoter fragment. The resulting constructs were designated pIG121.proHSPI, pIG121.proHSPII, and pIG121.proHSPIII. Promoter inserts were sequence-verified at the pUC57 cloning stage, while recombinant pIG121.Hm constructs were confirmed by restriction analysis. The original experimental record explicitly describes initial cloning, amplification, and sequencing in pUC57 followed by transfer of the promoter fragments into HindIII/BamHI-linearized pIG121.Hm. Moreover, the recombinant plasmids were propagated in *Escherichia coli* DH5α and subsequently introduced into *Agrobacterium tumefaciens* strain EHA101, a disarmed strain derived from the hypervirulent A281 background [38]. Transformed *Agrobacterium* colonies were selected on LB medium containing kanamycin (50 mg L^−1^), and positive colonies were confirmed by colony PCR using promoter/*GUS*-specific primers. For the colony plate shown in Figure S4, LB medium contained kanamycin and carbenicillin at 50 mg L^−1^ each. 

### 4.6. Generation and Molecular Confirmation of Transgenic Brassica napus

*Brassica napus* cv. Bulbul-98 was used as the heterologous system for functional promoter analysis. Surface-sterilized seeds were germinated, and one-week-old seedlings were transferred to pots and acclimatized before transformation. *A. tumefaciens* EHA101 harboring the respective promoter::*GUS* constructs was cultured at 28 °C. Cells were harvested and resuspended in half-strength Murashige and Skoog medium supplemented with 3% sucrose and 0.5 mg L^−1^ 6-benzylaminopurine (BAP). Murashige and Skoog medium remains the foundational basal medium for plant tissue and transformation procedures [40]. 

An *in planta* wound-inoculation approach was used for transformation, consistent with the broader application of *in planta* transformation strategies in *B. napus* [41,42]. At the transformation stage, the primary shoot was cut near its base, leaving two leaves, and the *Agrobacterium* suspension was applied directly to the wounded region using a micropipette. Secondary shoots emerging from the treated region were allowed to develop and flower. Genomic DNA was isolated from tissue derived from the emerging secondary shoots, and putative transformants were screened by PCR. PCR-positive primary transformants were grown to maturity, and their seeds were subjected to kanamycin selection to obtain the T_1_ generation. Seeds harvested from individual T_1_ plants were subsequently germinated and propagated to obtain T_2_ progeny. T_2_ plants were screened by genomic PCR using GUS-F/R primers (Table S6). PCR-positive T_2_ plants were subsequently used for promoter activity analyses. Briefly, throughout the transformation and selection process, seeds and progeny derived from individual T_0_ parental plants were maintained separately through the T_1_ and T_2_ generations, without pooling material from different parental lineages. For each promoter construct, three biological replicates derived from three different T_0_ parental plants and their respective T_2_ descendants were used for promoter-expression analysis. This approach preserved the identity of each parental lineage throughout the experimental process.

### 4.7. Functional Analysis of Promoter Deletions and Stress-Responsive GUS Expression

To compare heat-responsive activity among the three promoter fragments, PCR-confirmed transgenic *B. napus* plants carrying proHSPI::*GUS*, proHSPII::*GUS*, or proHSPIII::*GUS* were exposed to 42 °C and sampled after 0, 3, 6, 12, and 24 h. Preliminary time-course analysis showed the strongest reporter transcript accumulation at 6 h; therefore, this time point was selected for direct comparison of the three promoter constructs. The *GUS* transcript abundance was determined by semi-quantitative RT-PCR using GUS-F/R primers. *B. napus Actin* (GenBank accession AF111812) was used as the internal reference, with *BnActin*-F/R primers. The respective primer sequences are provided in Table S6. Reporter signals were normalized to the corresponding *Actin* signal and used to compare the relative activity of proHSPI, proHSPII, and proHSPIII under heat stress. To further quantify reporter-gene expression, quantitative real-time PCR (RT-qPCR) was performed using transgenic B. napus plants carrying proHSPI::*GUS*, proHSPII::GUS, or proHSPIII::GUS under unstressed control and heat-stress conditions. *BnActin* was used as the reference gene, and three biological replicates were analyzed for each construct and condition. Relative GUS transcript abundance was calculated using the comparative 2^−ΔΔ*Cq*^ method. Briefly, Δ*Cq* was calculated by subtracting the *BnActin Cq* value from the corresponding *GUS Cq* value, and ΔΔ*Cq* was determined relative to the designated unstressed control. Relative *GUS* expression was then calculated as 2^−ΔΔ*Cq*^. The pair of primer sequences used for RT-qPCR are provided in Table S6. The −1012 bp proHSPII fragment retained strong heat-responsive activity; therefore, it was selected for further analysis under individual and combined abiotic stresses. PCR-confirmed T_2_ proHSPII::*GUS* plants were subjected to heat, drought, or simultaneous heat and drought treatments. For heat stress, plants were exposed to 42 °C and sampled at 0, 1, 3, and 6 h. Drought was imposed by withholding water, with samples collected at 0, 3, 6, 12, and 24 h. For combined stress, water was withheld while plants were simultaneously exposed to 42 °C, with samples collected at the same 0, 3, 6, 12, and 24 h time points. Total RNA was extracted from each sample and converted to cDNA as described above. *GUS* transcripts were amplified by semi-quantitative RT-PCR and normalized to *BnActin*. For individual time-course experiments, normalized reporter transcript abundance was expressed relative to the corresponding untreated 0 h sample.

### 4.8. Densitometric and Statistical Analyses

Semi-quantitative RT-PCR band intensities were quantified using ImageJ (Version 1.54t, 2026). Background correction was applied during band-intensity quantification to minimize the contribution of nonspecific background signals. Target-gene or reporter signals were normalized to the corresponding *Actin* signal for each biological replicate. For time-course experiments, normalized values were expressed relative to the respective untreated control unless otherwise indicated. Data are presented as the mean ± standard deviation (SD) of three biological replicates (n = 3). Statistical analyses were performed using STATISTIX 9.0 (Analytical Software, Tallahassee, FL, USA). Differences among treatment time points or promoter constructs were evaluated by one-way analysis of variance (ANOVA), followed by Tukey’s honestly significant difference (HSD) test for multiple comparisons. For RT-qPCR data comparing promoter constructs and treatment conditions (normal, unstressed conditions and heat stress), two-way ANOVA followed by an appropriate post hoc multiple-comparison test was used. Differences were considered statistically significant at *p* < 0.05. Different lowercase letters indicate statistically significant differences among groups, whereas groups sharing one or more letters are not significantly different.

## 5. Conclusions

This study characterizes the stress-responsive expression of *ClHSP22.9* and identifies an important regulatory region within its promoter. Native *ClHSP22.9* transcript abundance increased in response to heat, dehydration, and salinity, whereas cold treatment elicited a comparatively limited transcriptional response. Serial 5′ deletion analysis identified the −1012 to −522 bp region as an important contributor to maximal heat-responsive promoter activity. Furthermore, the −1012 bp proHSPII fragment retained stress-responsive *GUS* transcription in transgenic *Brassica napus* under heat, drought, and combined heat–drought conditions. These findings establish proHSPII as a promising candidate stress-inducible regulatory sequence for further characterization and potential application in plant biotechnology. However, the contributions of individual *cis*-regulatory elements, direct *GUS* enzymatic activity, and promoter responsiveness to cold and salinity remain to be established.

## Data Availability

The original contributions presented in this study are included in the article/Supplementary Material. Further inquiries can be directed to the corresponding authors.

## Supplementary Materials

The following supporting information can be downloaded at: https://www.mdpi.com/article/10.3390/ijms27219648/s1.

## Abbreviations

The following abbreviations are used in this manuscript:

sHSPs	small heat shock proteins
HSEs	heat shock elements
GUS	β-glucuronidase
TAIL-PCR	thermal asymmetric interlaced PCR
ABREs	ABA-responsive elements
TSS	transcription start site
CaMV	cauliflower mosaic virus
ANOVA	Analysis of Variance
M-MLV	Moloney Murine Leukemia Virus
