# Supplementary material for: Functional Characterization of ClHSP22.9 Promoter from Wild Watermelon (Citrullus lanatus) Reveals Heat- and Drought-Inducible Transcriptional Activity
Source: Int J Mol Sci. 2026 Oct 28;27(21):9648. doi: 10.3390/ijms27219648 (PMC13762559; doi:10.3390/ijms27219648)
Supplement: Supplementary file 1 [file ijms-27-09648-s001.zip › Supplementary Figures.pdf]

Supplementary Figures

Functional Characterization of *ClHSP22.9* Promoter from Wild Watermelon (*Citrullus lanatus*) Reveals Heat- and Drought-Inducible Transcriptional Activity

Raheel Munir [1,2*], Iqbal Hussain [1], Nazia Jan [1], Bahar Ali [1,2], Iqbal Munir [2], Waqar Ali [2], Raham Sher Khan [3] and Aqib Iqbal [2,3*]

[1] Department of Horticulture, Zhejiang University, Hangzhou, 310058, China

[2] Institute of Biotechnology and Genetic Engineering, The University of Agriculture, Peshawar, 25130, Pakistan

[3] Department of Biotechnology, Abdul Wali Khan University, Mardan, 23200, Pakistan

[*] Correspondence: raheelmunir@zju.edu.cn; aqib@awkum.edu.pk

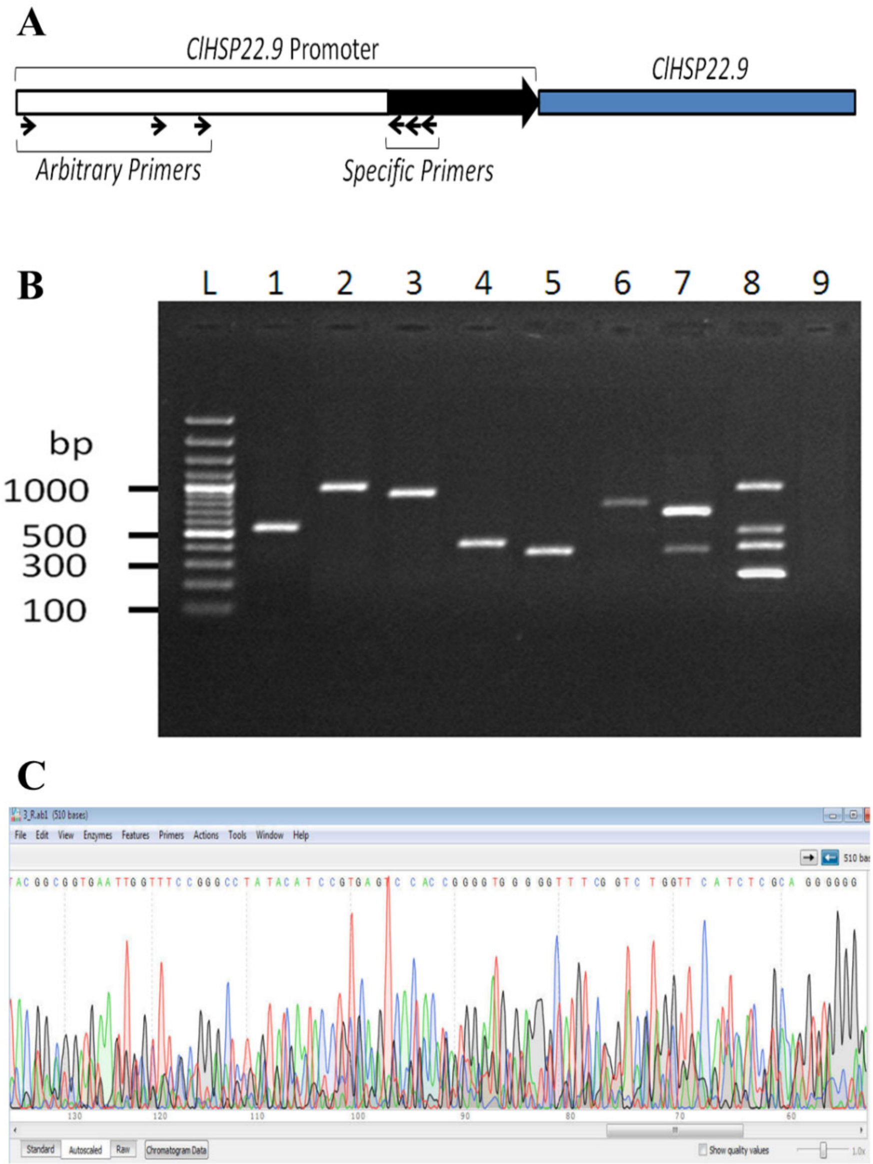

Figure S1. Isolation and sequence verification of the *ClHSP22.9* promoter from wild watermelon. (A) Schematic representation of the genome-walking strategy used to isolate the upstream regulatory region of *ClHSP22.9*, showing the relative positions of arbitrary primers and nested gene-specific primers. **(B)** Representative agarose gel electrophoresis of TAIL-PCR and partially overlapping primer-based PCR (POP-PCR) products. L, 100-bp DNA ladder; lane 1, gel-purified TAIL-PCR product; lanes 2 and 3, amplification products obtained with POP1/proHSP.G1 and POP1/proHSP.G2, respectively; lanes 4 and 5, products obtained with POP2/proHSP.G1 and POP2/proHSP.G2; lanes 6 and 7, products obtained with POP3/proHSP.G1 and POP3/proHSP.G2; lane 8, representative product of the primary PCR; lane 9, negative control. **(C)** Representative Sanger sequencing chromatogram of a purified POP-PCR product used for verification and assembly of the *ClHSP22.9* promoter sequence.

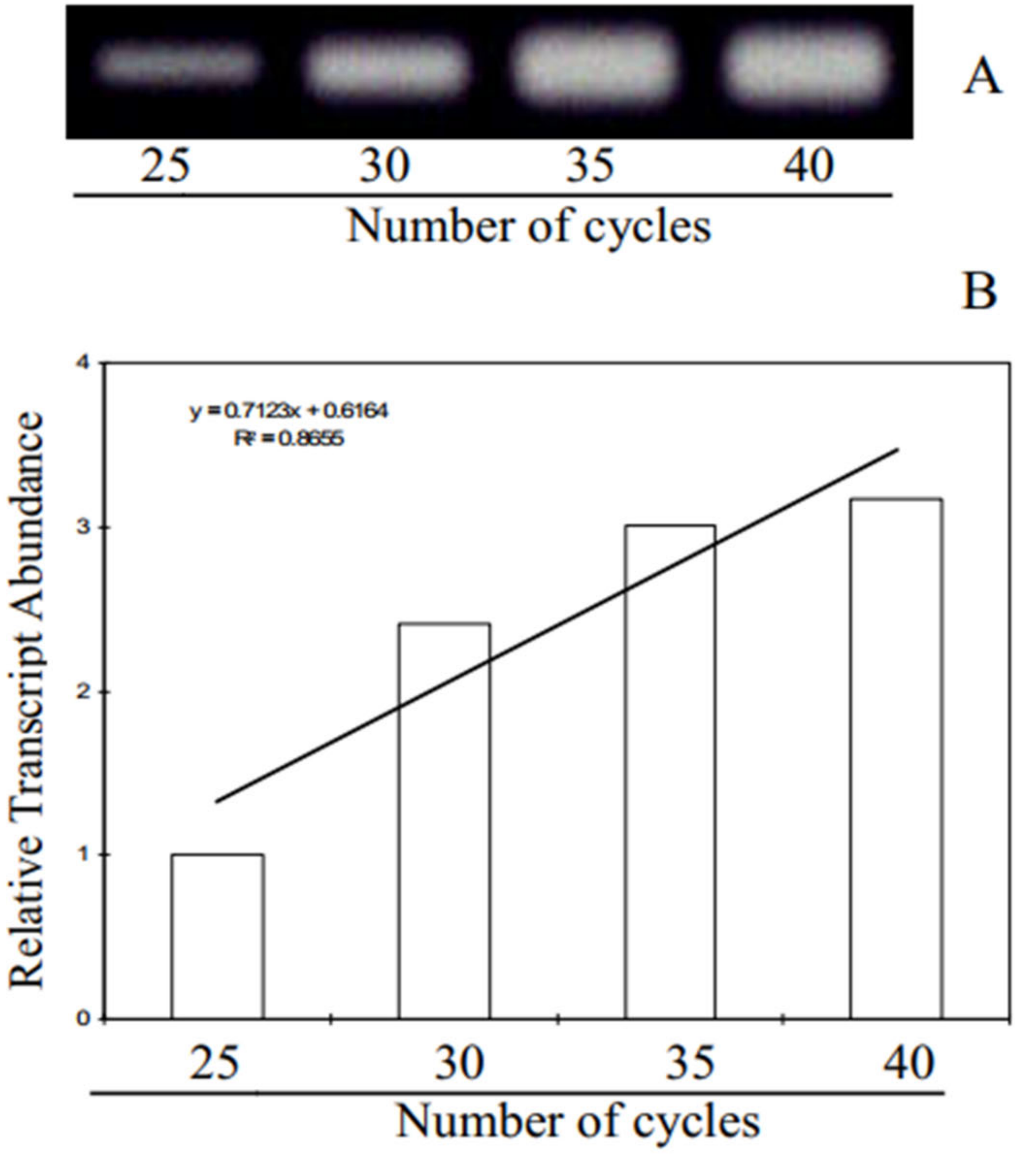

Figure S2. Optimization of PCR cycle number for semi-quantitative RT-PCR analysis of *ClHSP22.9*. (A) Representative agarose gel showing amplification of *ClHSP22.9* after 25, 30, 35, and 40 PCR cycles. **(B)** Corresponding densitometric analysis of the amplified products showing the increase in *ClHSP22.9* band intensity with increasing cycle number. Thirty amplification cycles were selected for subsequent semi-quantitative RT-PCR analyses to ensure readily detectable amplification while minimizing progression toward the plateau phase.

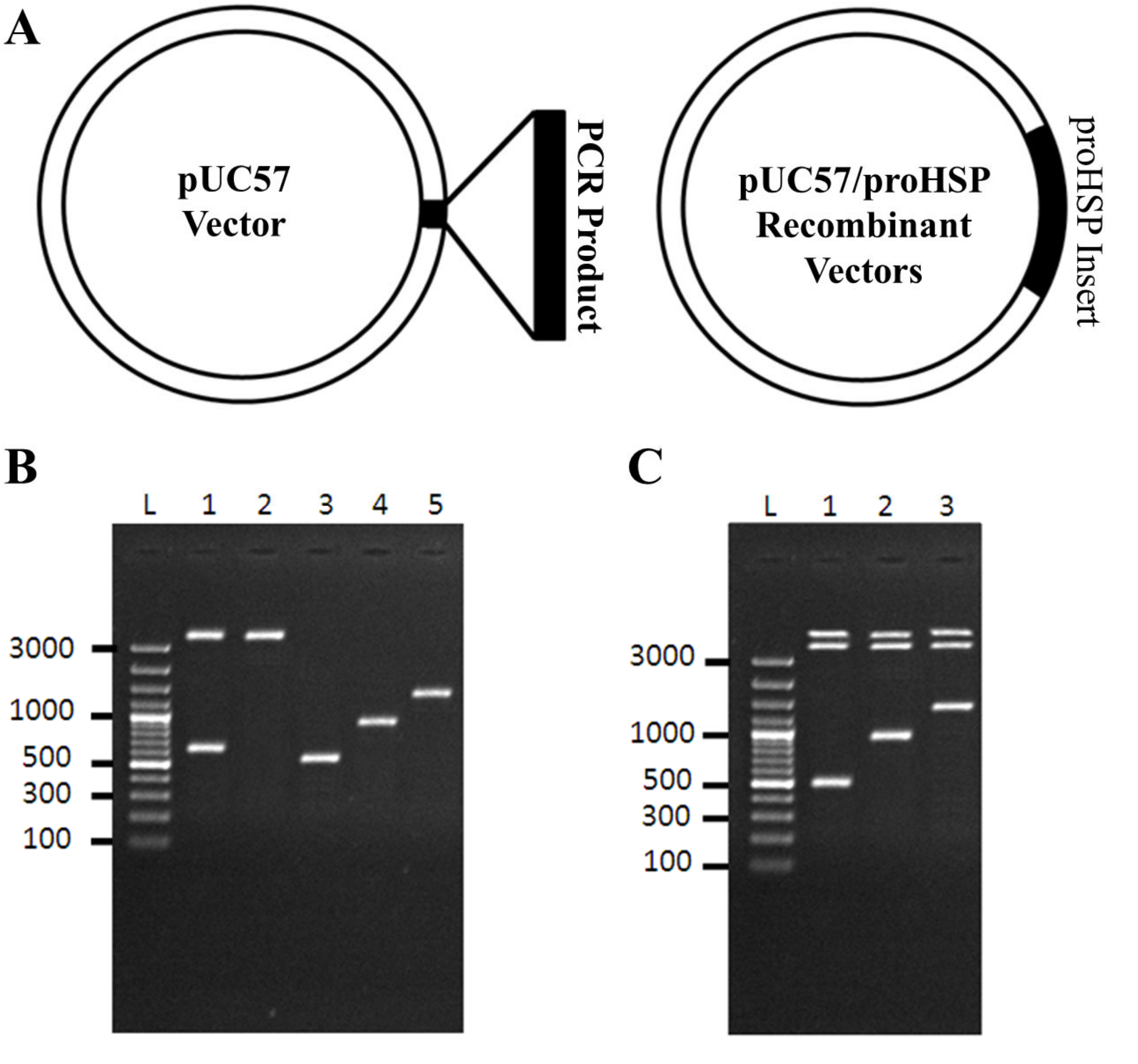

Figure S3. Initial cloning and molecular verification of *ClHSP22.9* promoter deletion fragments in the pUC57 vector. **(A)** Schematic representation of the cloning strategy used to insert the amplified *ClHSP22.9* promoter fragments into pUC57. **(B)** Agarose gel electrophoresis showing preparation of the vector and promoter fragments. L, 100bp DNA ladder; lane 1, restriction-digested pUC57-derived fragment; lane 2, linearized pUC57 vector; lanes 3-5, PCR-amplified and gel-purified proHSPIII (522 bp), proHSPII (1012 bp), and proHSPI (1482 bp) promoter fragments, respectively. **(C)** Restriction-digestion analysis of recombinant pUC57 constructs confirming insertion of proHSPIII, proHSPII, and proHSPI in lanes 1-3, respectively. The upper bands correspond to the pUC57 vector backbone, whereas the lower bands correspond to the released promoter inserts.

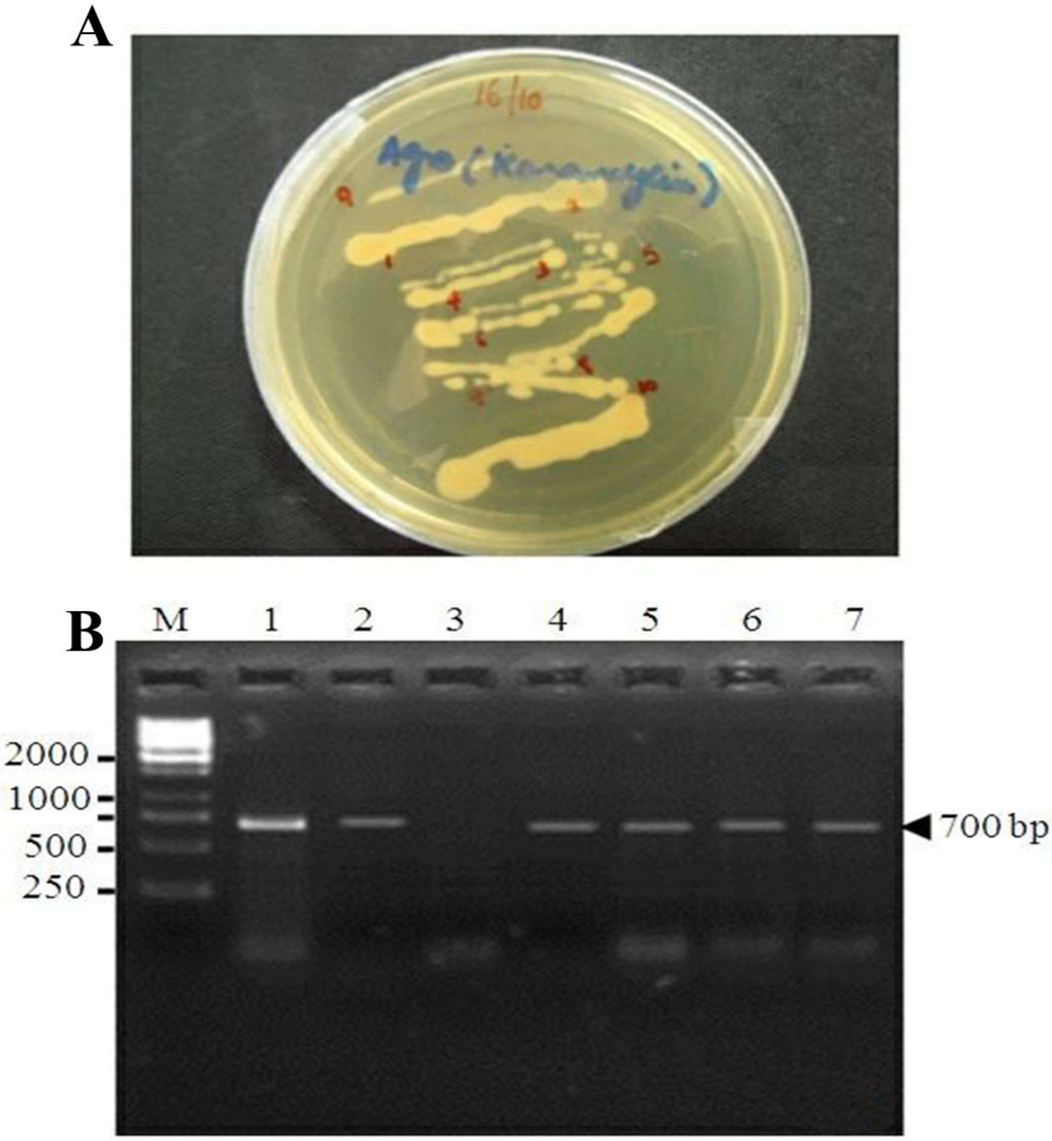

Figure S4. Molecular verification of *Agrobacterium* colonies harboring the *ClHSP22.9* promoter::*GUS* construct. (A) Representative *Agrobacterium* colonies grown on LB agar supplemented with kanamycin (50 mg L^{-1}) and carbenicillin (50 mg L^{-1}) following transformation with the recombinant promoter::*GUS* construct. **(B)** Colony PCR analysis confirming the presence of the recombinant construct in transformed *Agrobacterium*. M, 1-kb DNA ladder; lane 1, recombinant plasmid DNA used as a positive control; lanes 2-7, representative *Agrobacterium* colonies. The expected amplification product of approximately 700 bp is indicated by the arrow.

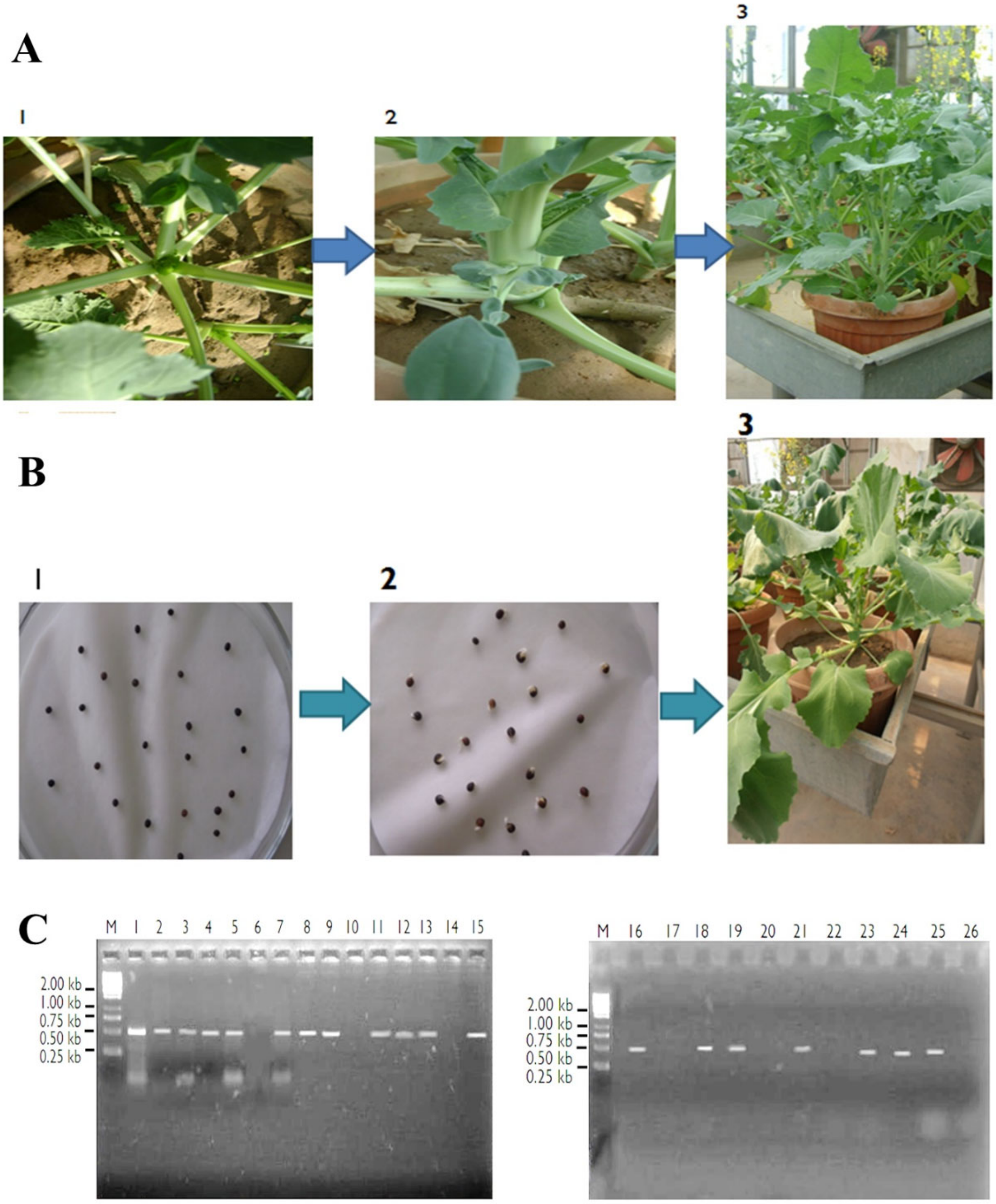

Figure S5. Generation, selection, and molecular confirmation of transgenic *Brassica napus* plants carrying the *ClHSP22.9* promoter::*GUS* construct. (A) Representative stages of the *in planta* transformation procedure, including application of *Agrobacterium* suspension to wounded plants (1), emergence and growth of secondary shoots from the treated region (2), and establishment of regenerated plants (3). **(B)** Selection of putative transgenic progeny, showing seed germination on kanamycin-containing medium (1), development of resistant seedlings (2), and subsequent growth of selected plants in pots (3). **(C)** PCR analysis of genomic DNA from T2 progeny using *GUS*-specific primers to confirm integration and inheritance of the transgene. M, molecular size marker; numbered lanes represent individual T2 plants analyzed for the presence of the *GUS* transgene.
