# Supplementary material for: Functional Characterization of ClHSP22.9 Promoter from Wild Watermelon (Citrullus lanatus) Reveals Heat- and Drought-Inducible Transcriptional Activity
Source: Int J Mol Sci. 2026 Oct 28;27(21):9648. doi: 10.3390/ijms27219648 (PMC13762559; doi:10.3390/ijms27219648)
Supplement: Supplementary file 1 [file ijms-27-09648-s001.zip › Tables - Supplementary Materials - S1-S4 & S6.pdf]

Supplementary Tables

Functional Characterization of *ClHSP22.9* Promoter from Wild Watermelon (*Citrullus lanatus*) Reveals Heat- and Drought-Inducible Transcriptional Activity

Raheel Munir[1,2*], Iqbal Hussain[1], Nazia Jan[1], Bahar Ali[1,2], Iqbal Munir[2], Waqar Ali[2], Raham Sher Khan[3] and Aqib Iqbal[2,3*]

[1] Department of Horticulture, Zhejiang University, Hangzhou, 310058, China

[2] Institute of Biotechnology and Genetic Engineering, The University of Agriculture, Peshawar, 25130, Pakistan

[3] Department of Biotechnology, Abdul Wali Khan University, Mardan, 23200, Pakistan

[*] Correspondence: raheelmunir@zju.edu.cn; aqib@awkum.edu.pk

Table S1. Primers used for semi-quantitative RT-PCR analysis of *ClHSP22.9* transcript abundance in wild watermelon

Primer	Sequence (5′–3′)	Target/Application	Accession no.
ClHSP22.9-F	GGATTCATGGCGATGATGGGCTTACCTAG	Amplification of *ClHSP22.9* transcript	HQ681891
ClHSP22.9-R	GAATCCCTTTCAGCCTTGGTAGGC	Amplification of *ClHSP22.9* transcript	HQ681891
Actin2/7-F	CATTCTCCGTTTGGACCTTGCT	Internal reference gene	GU565958
Actin2/7-R	TCGTAGTTTTCTCAATGGAGGAACTG	Internal reference gene	GU565958

Table S2. Primers used for TAIL-PCR and partially overlapping primer-based PCR (POP-PCR) isolation and confirmation of the *ClHSP22.9* promoter.

Primer Set	PCR Round	Sequence (5′ - 3′)
**ClHSP22.9-GPS1**	Primary promoter walking PCR	**GAAAAACAAAAACAGTAAGAATCCA**
**ClHSP22.9-GPS2**	Secondary nested PCR	**AACAGCAGGTTTCTAGGTAAGCCCA**
**ClHSP22.9-GPS3**	Tertiary nested PCR	**TTAATTTATGTTTTTTAGGGTACAT**
**Arbitrary Degenerate**		
**AD-1**	TAIL-PCR arbitrary primers	**AGTCGACAGATATGAA**
**AD-2**		**GTCCGAGACACAAGTT**
**AD-3**		**TGTGGAGTAGCATAGA**
**POP1**	Primary	AGTCAGCGTCCAGGTAGTCAGTCTC
	Secondary	TCAGGTCCAAGGTCAAGTCAGTCTC
	Tertiary	CTCAGCGTGTTCGTCAGTCAGTCTC
**POP2**	Primary	CAGTCAGTCTCAGGTCGTCTCCAGT
	Secondary	TCAGTCAGTCAGTTGCGTCTCCAGT
	Tertiary	AGCAGGTCAGTTACACGTCTCCAGT
**POP3**	Primary	CGCTTCAGATGGTACAGTGCAGTCA
	Secondary	ACACGATCCCAAGGTAGTGCAGTCA
	Tertiary	GTTACTCAGGTCCCAAGTGCAGTCA

Table S3: Thermal cycling conditions used for TAIL-PCR isolation of *ClHSP22.9* promoter.

Reaction	Number of Cycles	Thermal Conditions
Primary	1	95°C
	5	94°C (15 s), 63°C (1 min), 72°C (2
	1	min)
	10	94°C (15 s), 33°C (1 min), 72°C (2
	12	min)
		94°C (15 s), 44°C (1 min), 72°C (2
		min)
	1	94°C (5 s), 63°C (1 min), 72°C (2 min)
		94°C (5 s), 63°C (1 min), 72°C (2 min)
		94°C (5 s), 44°C (1 min), 72°C (2 min)
		72°C (10 min)
Secondary	10	94°C (5 s), 63°C (1 min), 72°C (2 min)
		94°C (5 s), 63°C (1 min), 72°C (2 min)
		94°C (5 s), 44°C (1 min), 72°C (2 min)
	1	72°C (5 min)
Tertiary		94°C (10 s), 44°C (1 min), 72°C (2
		min)
		72°C (5 min)

Table S4: Thermal cycling conditions used for partially overlapping primer-based PCR (POP PCR) isolation of ClHSP22.9 promoter.

Round of PCR	Stage	Thermal condition	Cycle number
Primary		94ºC 1 min, 98°C 1 min	1
	1	94ºC 30 s, 65°C 1 min, 72°C 2 min	5
	2	94ºC 30 s, 25°C 1 min, 72°C 2 min	1
	3	94ºC 30 s, 65°C 1 min, 72°C 2 min	30
		72ºC 5 min	1
1 µL of the product was directly used as template in the secondary round of PCR			
Secondary	1	94ºC 30 s, 65°C 1 min, 72°C 2 min	5
	2	94ºC 30 s, 50°C 1 min, 72°C 2 min	1
	3	94ºC 30 s, 65°C 1 min, 72°C 2 min	30
		72ºC 5 min	1
1 µL of the product was directly used as template in the tertiary round of PCR			
Tertiary	Reaction profile of tertiary PCR is identical to that of secondary PCR		

Table S6. Primers used for construction and molecular analysis of *ClHSP22.9* promoter::*GUS* reporter constructs in transgenic *Brassica napus*.

Primer Set	PCR Application	Sequence
proHSPI-F	For preparing template for promoter analysis	5'-AAGCTTGTGTAAAGTTCACGTGAAAA-3'
proHSPII-F		5'- AAGCTTCTTCCATCATTTGGCGTTTTT -3'
proHSPIII-F		5'- AAGCTTTGAACCAAAAAGAAAGAGAAA -3'
proHSP-R		5'-GGATCCATTTGAGGATGGAGGAAG -3'
GUS-F	Detection of GUS gene and transcripts	5'-TATCAGCGCGAAGCTTTTAT-3'
GUS-R		5'- CAGTTGCAACCACCTGTTGAT 3'
BnActin-F	Normalization of transcript abundance in transgenic *B. napus*	5'-TGAAAGATGGCCGATGGTGA-3'
BnActin-R		5'-TAGCGTAGAGAGAAAGAACAGCCTG-3'
HSP22-F	For cloning and molecular analysis	5' TCTAGAATGGCGATGATGGGCTTACCTAG -3'
HSP22-R		5' GAATTCCTTTCAGCCTTGGTAGGC -3'
GUS-F		5' CATCAGCCGATTATCATCACC -3'
GUS-R		5' TCCCTTTCTTGTTACCGCC -3'

BnActin; *Brassica napus Actin* GenBank Accession No. AF111812
